# Supplementary material for: Hot spot prediction in protein-protein interactions by an ensemble system
Source: BMC Syst Biol. 2018 Dec 31;12(Suppl 9):132. doi: 10.1186/s12918-018-0665-8 (PMC6311905; doi:10.1186/s12918-018-0665-8)
Supplement: Supplementary file 2 — All relevant MATLAB code. A simple Matlab implement of our predictor. (ZIP 19,742 kb) [file 12918_2018_665_MOESM2_ESM.zip › Additional file 2/Read ASA/ASA website/dbMPIKT/4KROA.html]

 NetSurfP - Protein Surface Accessibility and Secondary Structure Predictions

  

|  |  |  |
| --- | --- | --- |
|  |  | NetSurfP - Protein Surface Accessibility and Secondary Structure Predictions  Technical University of Denmark |

  


---

```
# For publication of results, please cite:
# A generic method for assignment of reliability scores applied to solvent accessibility predictions.
# Bent Petersen, Thomas Nordahl Petersen, Pernille Andersen, Morten Nielsen and Claus Lundegaard
# BMC Structural Biology 2009, 9:51 doi:10.1186/1472-6807-9-51
#
# Column 1: Class assignment - B for buried or E for Exposed - Threshold: 25% exposure, but not based on RSA
# Column 2: Amino acid
# Column 3: Sequence name
# Column 4: Amino acid number
# Column 5: Relative Surface Accessibility - RSA
# Column 6: Absolute Surface Accessibility
# Column 7: Z-fit score for RSA prediction
# Column 8: Probability for Alpha-Helix
# Column 9: Probability for Beta-strand
# Column 10: Probability for Coil
E L  4KROA                  1    0.679 124.270  -0.737   0.003   0.003   0.994
E E  4KROA                  2    0.618 108.000  -0.068   0.018   0.019   0.964
E E  4KROA                  3    0.665 116.228   0.632   0.018   0.047   0.935
E K  4KROA                  4    0.506 104.064   0.166   0.018   0.047   0.935
E K  4KROA                  5    0.511 105.092   1.248   0.019   0.141   0.840
B V  4KROA                  6    0.251  38.548   0.498   0.022   0.359   0.619
B C  4KROA                  7    0.056   7.876  -0.335   0.021   0.451   0.528
E Q  4KROA                  8    0.514  91.711   0.710   0.022   0.359   0.619
B G  4KROA                  9    0.174  13.694  -4.131   0.021   0.279   0.699
E T  4KROA                 10    0.381  52.817  -0.372   0.020   0.205   0.775
E S  4KROA                 11    0.549  64.378  -1.080   0.018   0.088   0.893
E N  4KROA                 12    0.424  62.044  -1.736   0.018   0.019   0.964
E K  4KROA                 13    0.579 119.121  -1.437   0.018   0.047   0.935
E L  4KROA                 14    0.322  59.013  -1.692   0.018   0.088   0.893
B T  4KROA                 15    0.212  29.363  -2.253   0.018   0.088   0.893
E Q  4KROA                 16    0.478  85.299  -1.325   0.018   0.088   0.893
B L  4KROA                 17    0.282  51.689  -2.715   0.018   0.047   0.935
E G  4KROA                 18    0.377  29.631  -2.201   0.018   0.019   0.964
E T  4KROA                 19    0.438  60.792  -0.931   0.058   0.017   0.925
E F  4KROA                 20    0.315  63.281  -1.013   0.430   0.016   0.555
E E  4KROA                 21    0.512  89.377  -0.884   0.430   0.016   0.555
E D  4KROA                 22    0.407  58.706  -1.405   0.354   0.048   0.598
E H  4KROA                 23    0.296  53.824   0.436   0.522   0.016   0.462
B F  4KROA                 24    0.203  40.782   0.415   0.879   0.010   0.111
E L  4KROA                 25    0.528  96.658   0.761   0.923   0.002   0.076
E S  4KROA                 26    0.405  47.419   0.226   0.923   0.002   0.076
B L  4KROA                 27    0.074  13.568  -0.276   0.923   0.002   0.076
E Q  4KROA                 28    0.280  50.026   0.740   0.923   0.002   0.076
E R  4KROA                 29    0.524 120.065   0.759   0.858   0.002   0.139
E M  4KROA                 30    0.268  53.527   0.934   0.782   0.003   0.216
B F  4KROA                 31    0.068  13.648  -0.033   0.522   0.016   0.462
E N  4KROA                 32    0.546  80.008   1.205   0.181   0.016   0.803
E N  4KROA                 33    0.586  85.820   0.253   0.018   0.019   0.964
B C  4KROA                 34    0.046   6.472   0.842   0.004   0.138   0.858
E E  4KROA                 35    0.311  54.402   0.541   0.003   0.718   0.279
B V  4KROA                 36    0.240  36.827   1.322   0.001   0.959   0.040
B V  4KROA                 37    0.081  12.480   0.761   0.001   0.959   0.040
B L  4KROA                 38    0.327  59.910  -0.104   0.002   0.816   0.182
B G  4KROA                 39    0.099   7.768  -1.108   0.003   0.718   0.279
B N  4KROA                 40    0.255  37.391  -0.143   0.002   0.816   0.182
B L  4KROA                 41    0.060  10.968   0.684   0.001   0.900   0.099
B E  4KROA                 42    0.206  36.023   0.381   0.001   0.959   0.040
B I  4KROA                 43    0.033   6.049   0.551   0.001   0.959   0.040
B T  4KROA                 44    0.109  15.118   0.933   0.001   0.900   0.099
B Y  4KROA                 45    0.115  24.490   0.151   0.002   0.816   0.182
B V  4KROA                 46    0.052   7.946   0.223   0.004   0.514   0.481
E Q  4KROA                 47    0.454  81.084  -0.320   0.019   0.141   0.840
E R  4KROA                 48    0.545 124.759  -1.111   0.018   0.047   0.935
B N  4KROA                 49    0.274  40.172  -0.956   0.018   0.047   0.935
E Y  4KROA                 50    0.451  96.400  -1.093   0.018   0.019   0.964
E D  4KROA                 51    0.549  79.140  -0.968   0.018   0.019   0.964
B L  4KROA                 52    0.154  28.197  -0.880   0.018   0.047   0.935
E S  4KROA                 53    0.494  57.944  -1.355   0.053   0.043   0.903
B F  4KROA                 54    0.244  48.891  -0.018   0.053   0.043   0.903
B L  4KROA                 55    0.116  21.166  -0.492   0.053   0.043   0.903
E K  4KROA                 56    0.560 115.130   0.405   0.053   0.043   0.903
E T  4KROA                 57    0.441  61.181   0.494   0.113   0.087   0.800
B I  4KROA                 58    0.106  19.628   0.459   0.066   0.296   0.638
E Q  4KROA                 59    0.340  60.778   0.477   0.087   0.683   0.230
E E  4KROA                 60    0.290  50.576   0.166   0.021   0.756   0.223
B V  4KROA                 61    0.081  12.450   0.566   0.018   0.846   0.136
B A  4KROA                 62    0.246  27.087   0.161   0.023   0.655   0.322
B G  4KROA                 63    0.118   9.255  -1.096   0.023   0.655   0.322
B Y  4KROA                 64    0.081  17.352   0.559   0.018   0.846   0.136
B V  4KROA                 65    0.031   4.749   0.201   0.011   0.918   0.071
B L  4KROA                 66    0.054   9.924   0.825   0.011   0.918   0.071
B I  4KROA                 67    0.027   4.976   0.529   0.018   0.846   0.136
B A  4KROA                 68    0.084   9.224  -0.556   0.021   0.756   0.223
B L  4KROA                 69    0.138  25.231  -0.283   0.022   0.552   0.426
B N  4KROA                 70    0.205  29.939  -0.524   0.020   0.205   0.775
E T  4KROA                 71    0.430  59.683  -0.466   0.018   0.088   0.893
B V  4KROA                 72    0.117  17.983  -0.668   0.018   0.088   0.893
E E  4KROA                 73    0.618 108.017  -0.011   0.019   0.141   0.840
E R  4KROA                 74    0.501 114.729   0.094   0.020   0.205   0.775
B I  4KROA                 75    0.175  32.375   0.976   0.020   0.205   0.775
E P  4KROA                 76    0.465  65.969  -0.409   0.018   0.088   0.893
B L  4KROA                 77    0.105  19.280  -0.297   0.018   0.047   0.935
E E  4KROA                 78    0.522  91.211  -0.114   0.018   0.019   0.964
E N  4KROA                 79    0.445  65.177  -0.185   0.018   0.047   0.935
B L  4KROA                 80    0.072  13.220   0.460   0.021   0.451   0.528
E Q  4KROA                 81    0.327  58.349   0.678   0.018   0.846   0.136
E I  4KROA                 82    0.260  48.081   0.272   0.011   0.918   0.071
B I  4KROA                 83    0.113  20.905   1.284   0.011   0.918   0.071
E R  4KROA                 84    0.332  76.005   0.614   0.023   0.655   0.322
B G  4KROA                 85    0.189  14.866  -2.239   0.021   0.279   0.699
E N  4KROA                 86    0.422  61.752   0.488   0.069   0.386   0.545
E M  4KROA                 87    0.486  97.229   0.453   0.021   0.756   0.223
E Y  4KROA                 88    0.269  57.507   0.662   0.087   0.683   0.230
B Y  4KROA                 89    0.227  48.531   0.106   0.074   0.484   0.442
E E  4KROA                 90    0.519  90.687  -1.056   0.125   0.227   0.648
E N  4KROA                 91    0.526  76.948  -1.307   0.056   0.142   0.802
E S  4KROA                 92    0.413  48.450   0.404   0.064   0.216   0.721
B Y  4KROA                 93    0.118  25.131  -0.123   0.021   0.451   0.528
B A  4KROA                 94    0.030   3.295  -0.316   0.021   0.756   0.223
B L  4KROA                 95    0.032   5.896  -0.177   0.011   0.918   0.071
B A  4KROA                 96    0.029   3.185   0.257   0.006   0.962   0.032
B V  4KROA                 97    0.019   2.951   0.881   0.006   0.962   0.032
B L  4KROA                 98    0.032   5.896  -0.209   0.006   0.962   0.032
B S  4KROA                 99    0.066   7.688  -0.982   0.006   0.962   0.032
B N  4KROA                100    0.087  12.722  -0.350   0.021   0.756   0.223
B Y  4KROA                101    0.234  49.963  -0.530   0.021   0.451   0.528
E D  4KROA                102    0.437  63.015  -0.839   0.021   0.279   0.699
E A  4KROA                103    0.532  58.659  -1.644   0.018   0.047   0.935
E N  4KROA                104    0.595  87.123  -2.185   0.018   0.047   0.935
E K  4KROA                105    0.472  97.090  -1.077   0.018   0.047   0.935
E T  4KROA                106    0.353  48.989  -1.318   0.018   0.088   0.893
E G  4KROA                107    0.346  27.238  -0.216   0.021   0.279   0.699
B L  4KROA                108    0.117  21.478  -0.742   0.022   0.552   0.426
E K  4KROA                109    0.315  64.754  -0.262   0.021   0.756   0.223
E E  4KROA                110    0.314  54.856  -0.277   0.021   0.756   0.223
B L  4KROA                111    0.056  10.199   1.172   0.021   0.756   0.223
E P  4KROA                112    0.379  53.752  -0.061   0.023   0.655   0.322
B M  4KROA                113    0.045   8.944  -0.375   0.005   0.262   0.733
E R  4KROA                114    0.379  86.814  -0.013   0.004   0.138   0.858
E N  4KROA                115    0.562  82.233  -0.605   0.018   0.088   0.893
B L  4KROA                116    0.209  38.231  -0.805   0.019   0.141   0.840
B Q  4KROA                117    0.233  41.560   0.016   0.022   0.359   0.619
E E  4KROA                118    0.242  42.208   0.061   0.021   0.756   0.223
B I  4KROA                119    0.040   7.492   0.241   0.018   0.846   0.136
B L  4KROA                120    0.155  28.362  -0.277   0.002   0.816   0.182
E H  4KROA                121    0.387  70.486  -0.949   0.004   0.616   0.381
B G  4KROA                122    0.203  15.945  -0.443   0.005   0.336   0.660
B A  4KROA                123    0.117  12.871  -1.319   0.004   0.514   0.481
B V  4KROA                124    0.044   6.794  -0.743   0.001   0.959   0.040
B R  4KROA                125    0.158  36.274   1.183   0.000   0.983   0.017
B F  4KROA                126    0.036   7.125  -0.876   0.001   0.959   0.040
B S  4KROA                127    0.119  13.900  -0.049   0.001   0.959   0.040
E N  4KROA                128    0.414  60.566  -0.430   0.004   0.616   0.381
B N  4KROA                129    0.127  18.549  -0.555   0.004   0.138   0.858
E P  4KROA                130    0.582  82.515  -0.907   0.018   0.047   0.935
E A  4KROA                131    0.496  54.615  -1.110   0.019   0.141   0.840
B L  4KROA                132    0.222  40.612  -1.475   0.019   0.141   0.840
B C  4KROA                133    0.273  38.385  -1.583   0.020   0.205   0.775
E N  4KROA                134    0.507  74.283  -1.531   0.020   0.205   0.775
E V  4KROA                135    0.370  56.900  -0.928   0.019   0.141   0.840
E E  4KROA                136    0.276  48.235  -0.710   0.019   0.141   0.840
E S  4KROA                137    0.535  62.725  -0.555   0.118   0.150   0.732
E I  4KROA                138    0.430  79.568  -0.987   0.113   0.087   0.800
E Q  4KROA                139    0.394  70.368  -0.676   0.115   0.016   0.868
B W  4KROA                140    0.117  28.114  -0.646   0.339   0.016   0.645
E R  4KROA                141    0.558 127.668   0.801   0.354   0.048   0.598
E D  4KROA                142    0.336  48.461   0.296   0.502   0.102   0.396
B I  4KROA                143    0.066  12.229  -0.842   0.502   0.102   0.396
B V  4KROA                144    0.188  28.972  -0.218   0.428   0.171   0.402
E S  4KROA                145    0.592  69.406   0.394   0.199   0.152   0.649
E S  4KROA                146    0.496  58.143  -0.777   0.018   0.019   0.964
E D  4KROA                147    0.700 100.812  -0.731   0.005   0.015   0.979
E F  4KROA                148    0.498  99.929  -1.444   0.005   0.045   0.951
E L  4KROA                149    0.522  95.578  -0.272   0.004   0.085   0.910
E S  4KROA                150    0.427  49.998  -0.726   0.005   0.262   0.733
E N  4KROA                151    0.494  72.263  -0.524   0.004   0.420   0.576
B M  4KROA                152    0.233  46.543  -0.440   0.004   0.514   0.481
E S  4KROA                153    0.437  51.170  -1.683   0.021   0.451   0.528
E M  4KROA                154    0.441  88.144  -1.786   0.022   0.359   0.619
E D  4KROA                155    0.502  72.309  -1.520   0.021   0.279   0.699
E F  4KROA                156    0.316  63.501  -1.679   0.022   0.359   0.619
E Q  4KROA                157    0.475  84.817  -1.410   0.019   0.141   0.840
E N  4KROA                158    0.571  83.550  -1.955   0.018   0.019   0.964
E H  4KROA                159    0.687 124.893  -1.144   0.018   0.019   0.964
E L  4KROA                160    0.648 118.722  -1.703   0.005   0.045   0.951
E G  4KROA                161    0.406  31.960   0.235   0.004   0.138   0.858
E S  4KROA                162    0.536  62.819   1.121   0.005   0.262   0.733
B C  4KROA                163    0.125  17.564  -1.740   0.021   0.279   0.699
E Q  4KROA                164    0.572 102.213   0.546   0.021   0.279   0.699
E K  4KROA                165    0.616 126.649   1.041   0.020   0.205   0.775
B C  4KROA                166    0.074  10.390  -1.816   0.018   0.088   0.893
E D  4KROA                167    0.507  73.030  -0.195   0.018   0.047   0.935
E P  4KROA                168    0.743 105.403  -0.876   0.018   0.047   0.935
E S  4KROA                169    0.534  62.620  -0.152   0.018   0.047   0.935
B C  4KROA                170    0.158  22.141  -1.069   0.018   0.088   0.893
E P  4KROA                171    0.639  90.731  -0.779   0.004   0.138   0.858
E N  4KROA                172    0.606  88.733  -0.618   0.004   0.197   0.799
E G  4KROA                173    0.325  25.570  -0.333   0.004   0.420   0.576
E S  4KROA                174    0.430  50.431  -0.228   0.004   0.616   0.381
B C  4KROA                175    0.142  19.909  -2.492   0.023   0.655   0.322
E W  4KROA                176    0.466 112.073  -1.248   0.021   0.451   0.528
E G  4KROA                177    0.388  30.567  -1.976   0.018   0.088   0.893
E A  4KROA                178    0.665  73.239  -1.599   0.018   0.047   0.935
E G  4KROA                179    0.543  42.726  -1.377   0.018   0.088   0.893
E E  4KROA                180    0.402  70.177  -0.767   0.019   0.141   0.840
E E  4KROA                181    0.503  87.874  -0.984   0.021   0.279   0.699
E N  4KROA                182    0.432  63.303  -0.000   0.022   0.359   0.619
B C  4KROA                183    0.110  15.444  -2.835   0.021   0.451   0.528
E Q  4KROA                184    0.415  74.065  -0.573   0.022   0.552   0.426
E K  4KROA                185    0.432  88.924  -1.306   0.021   0.451   0.528
E L  4KROA                186    0.347  63.627  -0.690   0.021   0.279   0.699
E T  4KROA                187    0.464  64.301  -1.141   0.021   0.279   0.699
E K  4KROA                188    0.614 126.300  -0.796   0.019   0.141   0.840
E I  4KROA                189    0.414  76.516  -1.523   0.019   0.141   0.840
E I  4KROA                190    0.454  84.027  -0.665   0.022   0.359   0.619
E C  4KROA                191    0.243  34.131  -1.183   0.022   0.359   0.619
E A  4KROA                192    0.359  39.507  -0.751   0.020   0.205   0.775
E Q  4KROA                193    0.624 111.393  -1.419   0.019   0.141   0.840
E Q  4KROA                194    0.531  94.783  -0.118   0.018   0.088   0.893
B C  4KROA                195    0.129  18.041  -1.422   0.018   0.047   0.935
E S  4KROA                196    0.558  65.339  -1.048   0.018   0.047   0.935
E G  4KROA                197    0.676  53.170  -1.628   0.018   0.047   0.935
E R  4KROA                198    0.470 107.653  -1.351   0.020   0.205   0.775
B C  4KROA                199    0.247  34.623  -1.404   0.022   0.359   0.619
E R  4KROA                200    0.405  92.676  -1.513   0.022   0.359   0.619
E G  4KROA                201    0.318  25.058  -1.722   0.005   0.262   0.733
E K  4KROA                202    0.569 117.126  -1.636   0.019   0.141   0.840
E S  4KROA                203    0.593  69.546  -1.790   0.018   0.047   0.935
E P  4KROA                204    0.606  85.977  -1.135   0.018   0.019   0.964
E S  4KROA                205    0.435  50.970  -1.122   0.018   0.047   0.935
E D  4KROA                206    0.434  62.568  -0.675   0.018   0.088   0.893
E C  4KROA                207    0.418  58.659  -0.078   0.020   0.205   0.775
B C  4KROA                208    0.188  26.409  -0.265   0.021   0.279   0.699
B H  4KROA                209    0.234  42.583   0.052   0.020   0.205   0.775
E N  4KROA                210    0.532  77.841  -0.603   0.053   0.043   0.903
E Q  4KROA                211    0.476  85.085   0.539   0.053   0.043   0.903
B C  4KROA                212    0.034   4.802  -1.549   0.052   0.084   0.864
E A  4KROA                213    0.413  45.524  -0.496   0.052   0.084   0.864
E A  4KROA                214    0.592  65.216  -0.851   0.052   0.084   0.864
E G  4KROA                215    0.450  35.407  -0.098   0.018   0.088   0.893
B C  4KROA                216    0.099  13.942  -1.963   0.020   0.205   0.775
E T  4KROA                217    0.560  77.672  -1.156   0.004   0.197   0.799
E G  4KROA                218    0.421  33.164  -0.980   0.005   0.045   0.951
E P  4KROA                219    0.453  64.337  -1.275   0.005   0.015   0.979
E R  4KROA                220    0.573 131.309  -0.242   0.003   0.003   0.994
E E  4KROA                221    0.578 100.924  -0.521   0.018   0.019   0.964
E S  4KROA                222    0.581  68.140  -0.591   0.018   0.019   0.964
E D  4KROA                223    0.426  61.358   1.123   0.052   0.084   0.864
B C  4KROA                224    0.055   7.666  -0.560   0.064   0.216   0.721
E L  4KROA                225    0.316  57.933   0.416   0.074   0.484   0.442
E V  4KROA                226    0.469  72.116   0.743   0.074   0.484   0.442
B C  4KROA                227    0.071   9.912  -0.754   0.066   0.296   0.638
E R  4KROA                228    0.463 105.912   0.153   0.066   0.296   0.638
E K  4KROA                229    0.508 104.413  -0.474   0.064   0.216   0.721
E F  4KROA                230    0.293  58.805  -0.809   0.066   0.296   0.638
E R  4KROA                231    0.415  94.989  -1.048   0.020   0.205   0.775
E D  4KROA                232    0.429  61.761  -1.353   0.019   0.141   0.840
E E  4KROA                233    0.645 112.629  -1.506   0.005   0.045   0.951
E A  4KROA                234    0.499  55.045  -1.996   0.005   0.045   0.951
E T  4KROA                235    0.449  62.221   0.148   0.005   0.336   0.660
B C  4KROA                236    0.083  11.625  -1.884   0.004   0.616   0.381
E K  4KROA                237    0.362  74.525   0.163   0.021   0.756   0.223
E D  4KROA                238    0.365  52.597  -1.233   0.021   0.756   0.223
E T  4KROA                239    0.513  71.111   0.288   0.021   0.451   0.528
B C  4KROA                240    0.057   8.003   0.157   0.005   0.045   0.951
E P  4KROA                241    0.441  62.620  -0.473   0.005   0.015   0.979
E P  4KROA                242    0.663  94.051  -1.103   0.018   0.019   0.964
E L  4KROA                243    0.450  82.432  -2.152   0.018   0.088   0.893
B M  4KROA                244    0.286  57.289  -0.693   0.021   0.451   0.528
B L  4KROA                245    0.230  42.113  -1.260   0.023   0.655   0.322
E Y  4KROA                246    0.419  89.540  -1.070   0.023   0.655   0.322
E N  4KROA                247    0.301  44.037  -1.458   0.005   0.262   0.733
E P  4KROA                248    0.670  95.059  -1.356   0.005   0.045   0.951
E T  4KROA                249    0.697  96.688  -1.049   0.004   0.138   0.858
E T  4KROA                250    0.449  62.249   0.082   0.004   0.420   0.576
E Y  4KROA                251    0.291  62.144   0.215   0.003   0.718   0.279
E Q  4KROA                252    0.435  77.673   1.051   0.018   0.846   0.136
B M  4KROA                253    0.112  22.391  -1.498   0.021   0.756   0.223
E D  4KROA                254    0.449  64.672  -0.202   0.023   0.655   0.322
E V  4KROA                255    0.623  95.694  -0.500   0.020   0.205   0.775
E N  4KROA                256    0.256  37.522  -0.510   0.018   0.019   0.964
E P  4KROA                257    0.625  88.673  -0.746   0.018   0.019   0.964
E E  4KROA                258    0.654 114.289  -0.306   0.018   0.047   0.935
E G  4KROA                259    0.386  30.347  -0.347   0.020   0.205   0.775
E K  4KROA                260    0.266  54.696  -1.395   0.022   0.359   0.619
E Y  4KROA                261    0.361  77.146  -0.274   0.021   0.451   0.528
E S  4KROA                262    0.495  57.991  -1.291   0.020   0.205   0.775
E F  4KROA                263    0.427  85.679  -1.041   0.004   0.085   0.910
E G  4KROA                264    0.610  47.968  -1.624   0.005   0.015   0.979
E A  4KROA                265    0.554  61.029  -0.919   0.005   0.015   0.979
E T  4KROA                266    0.656  90.973   0.398   0.018   0.088   0.893
E C  4KROA                267    0.237  33.219  -0.564   0.022   0.359   0.619
E V  4KROA                268    0.238  36.581  -0.440   0.022   0.552   0.426
E K  4KROA                269    0.491 101.040  -0.580   0.021   0.451   0.528
E K  4KROA                270    0.433  88.965  -0.528   0.022   0.359   0.619
B C  4KROA                271    0.109  15.360  -0.631   0.004   0.138   0.858
E P  4KROA                272    0.556  78.840  -1.348   0.005   0.045   0.951
E R  4KROA                273    0.655 150.087  -1.635   0.018   0.047   0.935
E N  4KROA                274    0.443  64.870  -1.594   0.019   0.141   0.840
B Y  4KROA                275    0.183  39.214  -0.690   0.022   0.359   0.619
B V  4KROA                276    0.273  41.914  -0.934   0.022   0.552   0.426
B V  4KROA                277    0.252  38.702  -0.671   0.023   0.655   0.322
E T  4KROA                278    0.312  43.344  -0.793   0.021   0.451   0.528
E D  4KROA                279    0.565  81.417  -1.878   0.019   0.141   0.840
E H  4KROA                280    0.671 122.109  -1.682   0.005   0.045   0.951
E G  4KROA                281    0.457  35.950  -0.654   0.004   0.138   0.858
E S  4KROA                282    0.350  41.020   0.458   0.005   0.336   0.660
B C  4KROA                283    0.081  11.302  -1.494   0.023   0.655   0.322
B V  4KROA                284    0.239  36.780  -0.290   0.021   0.756   0.223
E R  4KROA                285    0.371  84.959  -0.699   0.023   0.655   0.322
E A  4KROA                286    0.474  52.235  -0.116   0.021   0.451   0.528
B C  4KROA                287    0.080  11.232  -1.406   0.018   0.088   0.893
E G  4KROA                288    0.549  43.198  -1.329   0.005   0.045   0.951
E A  4KROA                289    0.678  74.771  -1.817   0.018   0.047   0.935
E D  4KROA                290    0.540  77.828  -2.092   0.018   0.047   0.935
E S  4KROA                291    0.314  36.789  -1.349   0.021   0.279   0.699
E Y  4KROA                292    0.312  66.568  -0.915   0.022   0.552   0.426
E E  4KROA                293    0.468  81.777  -0.762   0.023   0.655   0.322
E M  4KROA                294    0.296  59.210  -1.288   0.022   0.552   0.426
E E  4KROA                295    0.543  94.897  -1.447   0.021   0.279   0.699
E E  4KROA                296    0.698 121.976  -1.572   0.005   0.045   0.951
E D  4KROA                297    0.661  95.250  -0.691   0.018   0.019   0.964
E G  4KROA                298    0.538  42.341  -0.268   0.018   0.019   0.964
E V  4KROA                299    0.363  55.716  -0.318   0.019   0.141   0.840
E R  4KROA                300    0.429  98.355   0.050   0.021   0.451   0.528
E K  4KROA                301    0.489 100.587   1.429   0.023   0.655   0.322
B C  4KROA                302    0.049   6.922  -1.407   0.023   0.655   0.322
E K  4KROA                303    0.470  96.638   0.492   0.022   0.552   0.426
E K  4KROA                304    0.575 118.380   0.434   0.022   0.359   0.619
B C  4KROA                305    0.143  20.035  -1.228   0.019   0.141   0.840
E E  4KROA                306    0.565  98.653  -1.453   0.019   0.141   0.840
E G  4KROA                307    0.525  41.317  -1.268   0.019   0.141   0.840
E P  4KROA                308    0.490  69.545  -1.271   0.019   0.141   0.840
B C  4KROA                309    0.193  27.069  -2.251   0.019   0.141   0.840
E R  4KROA                310    0.472 108.065  -1.345   0.021   0.279   0.699
E K  4KROA                311    0.477  98.181  -1.627   0.021   0.451   0.528
E V  4KROA                312    0.370  56.823  -1.639   0.021   0.451   0.528
E C  4KROA                313    0.414  58.112  -1.816   0.022   0.359   0.619
E N  4KROA                314    0.527  77.167  -2.013   0.020   0.205   0.775
E G  4KROA                315    0.561  44.190  -1.751   0.019   0.141   0.840
E I  4KROA                316    0.449  83.028  -0.918   0.020   0.205   0.775
E G  4KROA                317    0.484  38.051  -1.431   0.021   0.451   0.528
B I  4KROA                318    0.252  46.564  -2.063   0.022   0.552   0.426
E G  4KROA                319    0.424  33.369  -1.665   0.023   0.655   0.322
E E  4KROA                320    0.315  55.013  -1.750   0.022   0.552   0.426
E F  4KROA                321    0.408  81.805  -1.333   0.022   0.359   0.619
E K  4KROA                322    0.454  93.470  -1.813   0.020   0.205   0.775
E D  4KROA                323    0.439  63.332  -1.867   0.019   0.141   0.840
E S  4KROA                324    0.435  51.029  -1.652   0.022   0.359   0.619
E L  4KROA                325    0.373  68.223  -1.356   0.022   0.552   0.426
E S  4KROA                326    0.334  39.168  -1.262   0.022   0.552   0.426
B I  4KROA                327    0.188  34.688  -1.613   0.021   0.451   0.528
E N  4KROA                328    0.421  61.590  -1.405   0.021   0.451   0.528
E A  4KROA                329    0.384  42.350  -1.418   0.021   0.279   0.699
E T  4KROA                330    0.385  53.358  -2.072   0.021   0.279   0.699
E N  4KROA                331    0.444  65.031  -1.982   0.021   0.279   0.699
B I  4KROA                332    0.231  42.791  -2.001   0.020   0.205   0.775
E K  4KROA                333    0.560 115.130  -1.393   0.019   0.141   0.840
E H  4KROA                334    0.468  85.184  -1.077   0.019   0.141   0.840
E F  4KROA                335    0.312  62.638  -1.587   0.018   0.047   0.935
E K  4KROA                336    0.602 123.790  -1.114   0.005   0.045   0.951
E N  4KROA                337    0.551  80.622  -0.430   0.004   0.085   0.910
B C  4KROA                338    0.094  13.127  -1.902   0.004   0.197   0.799
E T  4KROA                339    0.463  64.218  -0.619   0.004   0.616   0.381
E S  4KROA                340    0.462  54.170   0.109   0.002   0.816   0.182
B I  4KROA                341    0.184  34.022  -0.884   0.003   0.718   0.279
E S  4KROA                342    0.581  68.117  -1.915   0.004   0.514   0.481
E G  4KROA                343    0.429  33.754  -2.053   0.004   0.197   0.799
E D  4KROA                344    0.498  71.719  -1.665   0.005   0.262   0.733
B L  4KROA                345    0.227  41.600  -1.202   0.022   0.552   0.426
E H  4KROA                346    0.459  83.583  -1.297   0.023   0.655   0.322
B I  4KROA                347    0.213  39.331  -1.644   0.023   0.655   0.322
E L  4KROA                348    0.360  65.824  -1.223   0.022   0.359   0.619
B P  4KROA                349    0.278  39.491  -1.918   0.021   0.279   0.699
E V  4KROA                350    0.542  83.244  -1.775   0.020   0.205   0.775
E A  4KROA                351    0.483  53.183  -1.831   0.021   0.279   0.699
B F  4KROA                352    0.238  47.706  -1.445   0.021   0.451   0.528
E R  4KROA                353    0.543 124.347  -1.570   0.021   0.451   0.528
E G  4KROA                354    0.519  40.814  -2.311   0.021   0.279   0.699
E D  4KROA                355    0.528  76.099  -2.140   0.019   0.141   0.840
E S  4KROA                356    0.345  40.434  -1.797   0.018   0.088   0.893
E F  4KROA                357    0.368  73.858  -1.625   0.019   0.141   0.840
E T  4KROA                358    0.487  67.575  -1.413   0.022   0.359   0.619
E H  4KROA                359    0.348  63.392  -1.781   0.021   0.279   0.699
E T  4KROA                360    0.397  55.064  -1.814   0.004   0.197   0.799
E P  4KROA                361    0.417  59.215  -2.101   0.004   0.085   0.910
E P  4KROA                362    0.506  71.730  -1.668   0.018   0.047   0.935
E L  4KROA                363    0.381  69.816  -1.789   0.018   0.047   0.935
E D  4KROA                364    0.549  79.125  -1.255   0.053   0.043   0.903
E P  4KROA                365    0.377  53.454  -1.915   0.053   0.043   0.903
E Q  4KROA                366    0.538  96.033  -0.697   0.056   0.142   0.802
E E  4KROA                367    0.324  56.638  -0.785   0.066   0.296   0.638
E L  4KROA                368    0.349  63.975  -0.797   0.066   0.296   0.638
E D  4KROA                369    0.638  91.950  -0.587   0.064   0.216   0.721
E I  4KROA                370    0.314  58.034  -0.980   0.052   0.084   0.864
E L  4KROA                371    0.307  56.193  -0.844   0.018   0.019   0.964
E K  4KROA                372    0.649 133.540  -1.162   0.005   0.015   0.979
E T  4KROA                373    0.511  70.848   0.014   0.018   0.088   0.893
B V  4KROA                374    0.126  19.366  -0.579   0.022   0.359   0.619
E K  4KROA                375    0.519 106.655   0.185   0.004   0.514   0.481
E E  4KROA                376    0.514  89.743   0.018   0.023   0.655   0.322
B I  4KROA                377    0.139  25.770  -0.778   0.004   0.616   0.381
E T  4KROA                378    0.477  66.104  -1.369   0.004   0.420   0.576
E G  4KROA                379    0.449  35.336  -1.789   0.004   0.197   0.799
E F  4KROA                380    0.373  74.821  -1.441   0.005   0.262   0.733
B L  4KROA                381    0.211  38.597  -1.173   0.004   0.514   0.481
E L  4KROA                382    0.459  84.061  -1.012   0.023   0.655   0.322
B I  4KROA                383    0.166  30.673  -2.117   0.022   0.552   0.426
E Q  4KROA                384    0.387  69.100  -1.587   0.022   0.552   0.426
E A  4KROA                385    0.520  57.260  -1.592   0.022   0.552   0.426
E W  4KROA                386    0.333  79.990  -1.179   0.022   0.359   0.619
E P  4KROA                387    0.383  54.390  -1.590   0.019   0.141   0.840
E E  4KROA                388    0.569  99.334  -1.548   0.018   0.088   0.893
E N  4KROA                389    0.566  82.877  -1.320   0.018   0.088   0.893
B R  4KROA                390    0.188  42.983  -1.194   0.018   0.088   0.893
E T  4KROA                391    0.494  68.476  -1.415   0.019   0.141   0.840
E D  4KROA                392    0.344  49.642  -2.046   0.019   0.141   0.840
E L  4KROA                393    0.351  64.341  -1.204   0.018   0.088   0.893
E H  4KROA                394    0.554 100.718  -1.799   0.019   0.141   0.840
E A  4KROA                395    0.334  36.763  -1.309   0.019   0.141   0.840
B F  4KROA                396    0.172  34.581  -2.366   0.018   0.088   0.893
E E  4KROA                397    0.654 114.306  -0.772   0.018   0.047   0.935
E N  4KROA                398    0.541  79.276  -0.789   0.018   0.047   0.935
B L  4KROA                399    0.175  32.079  -1.092   0.018   0.088   0.893
E E  4KROA                400    0.515  89.970  -0.446   0.004   0.420   0.576
E I  4KROA                401    0.339  62.771  -0.307   0.023   0.655   0.322
B I  4KROA                402    0.246  45.436  -1.003   0.022   0.552   0.426
E R  4KROA                403    0.614 140.514  -1.213   0.022   0.359   0.619
E G  4KROA                404    0.443  34.880  -0.978   0.021   0.279   0.699
E R  4KROA                405    0.511 116.950  -0.323   0.022   0.359   0.619
E T  4KROA                406    0.449  62.221  -0.696   0.021   0.451   0.528
E K  4KROA                407    0.338  69.547  -0.783   0.022   0.359   0.619
E Q  4KROA                408    0.480  85.728  -1.182   0.020   0.205   0.775
E H  4KROA                409    0.609 110.813  -2.345   0.018   0.088   0.893
E G  4KROA                410    0.583  45.882  -2.356   0.018   0.088   0.893
E Q  4KROA                411    0.472  84.228  -1.519   0.021   0.279   0.699
E F  4KROA                412    0.304  60.912  -1.225   0.021   0.451   0.528
B S  4KROA                413    0.270  31.703  -2.093   0.022   0.552   0.426
B L  4KROA                414    0.245  44.860  -1.610   0.021   0.756   0.223
B A  4KROA                415    0.213  23.440  -1.825   0.018   0.846   0.136
B V  4KROA                416    0.285  43.835  -1.072   0.021   0.756   0.223
E V  4KROA                417    0.508  78.080  -1.839   0.023   0.655   0.322
E S  4KROA                418    0.420  49.259  -2.122   0.020   0.205   0.775
E L  4KROA                419    0.484  88.620  -1.666   0.018   0.047   0.935
E N  4KROA                420    0.537  78.617  -1.104   0.018   0.088   0.893
B I  4KROA                421    0.193  35.760  -1.207   0.021   0.279   0.699
E T  4KROA                422    0.382  52.983  -1.032   0.021   0.451   0.528
E S  4KROA                423    0.351  41.149  -0.945   0.021   0.451   0.528
B L  4KROA                424    0.276  50.481  -1.554   0.021   0.451   0.528
E G  4KROA                425    0.369  29.056  -1.072   0.021   0.279   0.699
E L  4KROA                426    0.583 106.711  -2.058   0.018   0.088   0.893
E R  4KROA                427    0.550 125.835  -1.848   0.018   0.047   0.935
E S  4KROA                428    0.530  62.081  -1.465   0.018   0.088   0.893
E L  4KROA                429    0.309  56.523  -0.609   0.020   0.205   0.775
E K  4KROA                430    0.415  85.283  -0.910   0.021   0.279   0.699
E E  4KROA                431    0.426  74.422  -0.586   0.021   0.451   0.528
B I  4KROA                432    0.101  18.722  -1.549   0.022   0.359   0.619
E S  4KROA                433    0.495  58.026  -1.025   0.022   0.359   0.619
E D  4KROA                434    0.485  69.917  -1.353   0.020   0.205   0.775
E G  4KROA                435    0.391  30.803  -2.463   0.018   0.047   0.935
E D  4KROA                436    0.416  59.874  -1.451   0.004   0.197   0.799
B V  4KROA                437    0.211  32.477  -1.483   0.003   0.718   0.279
B I  4KROA                438    0.168  31.080  -0.697   0.018   0.846   0.136
B I  4KROA                439    0.156  28.823  -1.439   0.018   0.846   0.136
B S  4KROA                440    0.290  34.011  -1.314   0.021   0.756   0.223
E G  4KROA                441    0.339  26.664  -1.287   0.021   0.451   0.528
B N  4KROA                442    0.149  21.872  -1.736   0.020   0.205   0.775
E K  4KROA                443    0.379  78.022  -1.247   0.020   0.205   0.775
E N  4KROA                444    0.412  60.302  -1.862   0.021   0.279   0.699
B L  4KROA                445    0.261  47.698  -1.609   0.020   0.205   0.775
B C  4KROA                446    0.227  31.857  -1.836   0.019   0.141   0.840
E Y  4KROA                447    0.385  82.189  -1.709   0.019   0.141   0.840
E A  4KROA                448    0.468  51.596  -1.357   0.020   0.205   0.775
E N  4KROA                449    0.450  65.836  -1.243   0.022   0.359   0.619
E T  4KROA                450    0.369  51.194  -1.476   0.021   0.279   0.699
E I  4KROA                451    0.389  71.947  -1.352   0.021   0.279   0.699
E N  4KROA                452    0.537  78.544  -0.815   0.020   0.205   0.775
B W  4KROA                453    0.248  59.596  -0.986   0.019   0.141   0.840
E K  4KROA                454    0.565 116.159  -1.165   0.019   0.141   0.840
E K  4KROA                455    0.486 100.011  -0.879   0.020   0.205   0.775
B L  4KROA                456    0.196  35.851  -1.511   0.022   0.359   0.619
E F  4KROA                457    0.440  88.268  -1.118   0.022   0.552   0.426
E G  4KROA                458    0.417  32.786  -0.909   0.021   0.451   0.528
E T  4KROA                459    0.430  59.710  -1.203   0.019   0.141   0.840
E S  4KROA                460    0.542  63.476  -1.327   0.018   0.088   0.893
E G  4KROA                461    0.620  48.770  -1.278   0.018   0.047   0.935
E Q  4KROA                462    0.506  90.372  -0.732   0.018   0.047   0.935
E K  4KROA                463    0.537 110.543  -0.661   0.019   0.141   0.840
B T  4KROA                464    0.204  28.281  -1.782   0.021   0.279   0.699
E K  4KROA                465    0.503 103.529  -0.563   0.022   0.359   0.619
E I  4KROA                466    0.295  54.667  -1.288   0.022   0.359   0.619
E I  4KROA                467    0.492  91.002  -1.291   0.021   0.279   0.699
E S  4KROA                468    0.413  48.462  -1.588   0.021   0.279   0.699
E N  4KROA                469    0.431  63.113  -0.837   0.020   0.205   0.775
E R  4KROA                470    0.403  92.402  -1.063   0.019   0.141   0.840
E G  4KROA                471    0.417  32.810  -0.518   0.056   0.142   0.802
E E  4KROA                472    0.512  89.446  -1.233   0.064   0.216   0.721
E N  4KROA                473    0.407  59.556  -1.048   0.064   0.216   0.721
E S  4KROA                474    0.481  56.420  -1.477   0.020   0.205   0.775
E C  4KROA                475    0.431  60.540  -0.959   0.021   0.279   0.699
E K  4KROA                476    0.550 113.156  -0.750   0.020   0.205   0.775
E A  4KROA                477    0.523  57.679  -0.858   0.019   0.141   0.840
E T  4KROA                478    0.411  56.936  -0.208   0.019   0.141   0.840
E G  4KROA                479    0.479  37.674  -1.242   0.019   0.141   0.840
E Q  4KROA                480    0.390  69.690  -0.805   0.020   0.205   0.775
E V  4KROA                481    0.461  70.917  -1.846   0.022   0.359   0.619
E C  4KROA                482    0.364  51.063  -1.192   0.022   0.359   0.619
E H  4KROA                483    0.419  76.125  -1.242   0.021   0.279   0.699
E A  4KROA                484    0.361  39.804  -1.700   0.020   0.205   0.775
E L  4KROA                485    0.537  98.306  -1.419   0.018   0.088   0.893
B C  4KROA                486    0.151  21.243  -2.452   0.018   0.088   0.893
E S  4KROA                487    0.537  62.948  -0.925   0.018   0.088   0.893
E P  4KROA                488    0.508  72.114  -0.946   0.019   0.141   0.840
E E  4KROA                489    0.590 103.038  -0.900   0.019   0.141   0.840
E G  4KROA                490    0.483  37.981  -0.553   0.004   0.197   0.799
B C  4KROA                491    0.096  13.408  -3.194   0.004   0.197   0.799
E W  4KROA                492    0.518 124.579  -0.814   0.004   0.138   0.858
E G  4KROA                493    0.394  31.008  -1.458   0.005   0.015   0.979
E P  4KROA                494    0.653  92.618  -2.053   0.003   0.003   0.994
E E  4KROA                495    0.605 105.606  -1.428   0.003   0.003   0.994
E P  4KROA                496    0.333  47.267  -0.831   0.005   0.015   0.979
E R  4KROA                497    0.551 126.248  -1.113   0.005   0.015   0.979
E D  4KROA                498    0.283  40.723   0.153   0.019   0.141   0.840
B C  4KROA                499    0.068   9.533  -3.091   0.021   0.279   0.699
B V  4KROA                500    0.261  40.146  -1.059   0.021   0.451   0.528
E S  4KROA                501    0.401  46.985  -0.832   0.022   0.359   0.619
B C  4KROA                502    0.063   8.789  -1.505   0.021   0.279   0.699
E R  4KROA                503    0.492 112.576  -1.124   0.020   0.205   0.775
E N  4KROA                504    0.484  70.784  -1.334   0.019   0.141   0.840
E V  4KROA                505    0.291  44.742  -1.945   0.019   0.141   0.840
E S  4KROA                506    0.400  46.927  -1.474   0.019   0.141   0.840
E R  4KROA                507    0.427  97.691  -0.878   0.021   0.279   0.699
E G  4KROA                508    0.481  37.894  -0.734   0.020   0.205   0.775
E R  4KROA                509    0.499 114.202  -1.054   0.019   0.141   0.840
E E  4KROA                510    0.428  74.754  -0.715   0.019   0.141   0.840
B C  4KROA                511    0.224  31.393  -1.686   0.019   0.141   0.840
E V  4KROA                512    0.483  74.283  -0.460   0.019   0.141   0.840
E D  4KROA                513    0.610  87.887  -0.390   0.019   0.141   0.840
E K  4KROA                514    0.533 109.535   0.587   0.019   0.141   0.840
B C  4KROA                515    0.038   5.265  -3.279   0.018   0.088   0.893
E N  4KROA                516    0.541  79.202  -1.114   0.019   0.141   0.840
E L  4KROA                517    0.510  93.363  -1.693   0.004   0.138   0.858
E L  4KROA                518    0.456  83.549  -2.015   0.004   0.085   0.910
E E  4KROA                519    0.507  88.503  -2.135   0.004   0.085   0.910
E G  4KROA                520    0.479  37.666  -2.265   0.004   0.085   0.910
E E  4KROA                521    0.524  91.508  -1.874   0.005   0.045   0.951
E P  4KROA                522    0.431  61.187  -1.831   0.020   0.205   0.775
E R  4KROA                523    0.370  84.753  -0.985   0.021   0.451   0.528
E E  4KROA                524    0.410  71.592  -1.332   0.022   0.552   0.426
B F  4KROA                525    0.225  45.117  -1.305   0.021   0.451   0.528
E V  4KROA                526    0.365  56.116  -1.111   0.021   0.279   0.699
E E  4KROA                527    0.636 111.074  -0.885   0.018   0.088   0.893
E N  4KROA                528    0.710 103.900  -0.517   0.018   0.019   0.964
E S  4KROA                529    0.485  56.783   0.178   0.018   0.019   0.964
E E  4KROA                530    0.450  78.685   1.491   0.022   0.359   0.619
B C  4KROA                531    0.079  11.078  -0.783   0.023   0.655   0.322
E I  4KROA                532    0.383  70.947   0.748   0.023   0.655   0.322
E Q  4KROA                533    0.516  92.158   0.637   0.021   0.451   0.528
B C  4KROA                534    0.064   8.972  -0.605   0.052   0.084   0.864
E H  4KROA                535    0.396  71.960   0.260   0.053   0.043   0.903
E P  4KROA                536    0.657  93.157  -0.234   0.115   0.016   0.868
E E  4KROA                537    0.460  80.432  -0.001   0.113   0.087   0.800
B C  4KROA                538    0.059   8.298  -1.411   0.064   0.216   0.721
E L  4KROA                539    0.415  75.987  -0.136   0.066   0.296   0.638
E P  4KROA                540    0.560  79.450  -0.786   0.020   0.205   0.775
E Q  4KROA                541    0.304  54.384  -0.914   0.019   0.141   0.840
E A  4KROA                542    0.591  65.128  -1.751   0.019   0.141   0.840
E M  4KROA                543    0.502 100.530  -1.166   0.004   0.085   0.910
E N  4KROA                544    0.511  74.810  -0.637   0.018   0.088   0.893
E I  4KROA                545    0.506  93.592  -0.946   0.019   0.141   0.840
E T  4KROA                546    0.409  56.687  -0.558   0.019   0.141   0.840
B C  4KROA                547    0.160  22.506  -3.211   0.020   0.205   0.775
E T  4KROA                548    0.576  79.947  -0.642   0.020   0.205   0.775
E G  4KROA                549    0.376  29.599  -1.785   0.005   0.045   0.951
E R  4KROA                550    0.556 127.416  -1.816   0.005   0.015   0.979
E G  4KROA                551    0.556  43.726  -0.482   0.005   0.015   0.979
E P  4KROA                552    0.429  60.804  -0.608   0.018   0.019   0.964
E D  4KROA                553    0.675  97.253  -0.678   0.018   0.019   0.964
E N  4KROA                554    0.420  61.532   0.961   0.018   0.047   0.935
B C  4KROA                555    0.057   7.961  -1.122   0.020   0.205   0.775
E I  4KROA                556    0.396  73.186  -0.248   0.022   0.359   0.619
E Q  4KROA                557    0.522  93.318   0.024   0.022   0.359   0.619
B C  4KROA                558    0.141  19.853  -0.555   0.020   0.205   0.775
E A  4KROA                559    0.477  52.543  -1.075   0.020   0.205   0.775
E H  4KROA                560    0.454  82.637  -1.087   0.021   0.279   0.699
B Y  4KROA                561    0.205  43.702  -0.337   0.022   0.359   0.619
E I  4KROA                562    0.418  77.367  -0.960   0.022   0.359   0.619
E D  4KROA                563    0.397  57.237  -1.379   0.019   0.141   0.840
E G  4KROA                564    0.616  48.463  -1.061   0.005   0.015   0.979
E P  4KROA                565    0.653  92.604  -1.343   0.003   0.003   0.994
E H  4KROA                566    0.446  81.109   0.787   0.021   0.279   0.699
B C  4KROA                567    0.057   8.059  -1.568   0.023   0.655   0.322
E V  4KROA                568    0.320  49.107   0.581   0.021   0.756   0.223
E K  4KROA                569    0.460  94.581  -0.310   0.021   0.756   0.223
E T  4KROA                570    0.508  70.404   1.296   0.022   0.552   0.426
B C  4KROA                571    0.052   7.245  -0.152   0.004   0.085   0.910
E P  4KROA                572    0.541  76.839   0.086   0.005   0.015   0.979
E A  4KROA                573    0.735  81.041  -0.170   0.005   0.015   0.979
E G  4KROA                574    0.501  39.421  -1.364   0.018   0.088   0.893
B V  4KROA                575    0.289  44.389  -0.281   0.004   0.514   0.481
E M  4KROA                576    0.347  69.435  -0.960   0.021   0.756   0.223
E G  4KROA                577    0.386  30.410  -1.260   0.023   0.655   0.322
E E  4KROA                578    0.382  66.753  -1.652   0.021   0.451   0.528
E N  4KROA                579    0.581  85.102  -1.875   0.020   0.205   0.775
E N  4KROA                580    0.554  81.091  -1.632   0.020   0.205   0.775
E T  4KROA                581    0.473  65.591  -0.365   0.021   0.451   0.528
E L  4KROA                582    0.298  54.564  -0.696   0.022   0.552   0.426
E V  4KROA                583    0.468  71.993  -0.734   0.023   0.655   0.322
E W  4KROA                584    0.324  77.898  -1.410   0.022   0.552   0.426
E K  4KROA                585    0.514 105.812  -0.429   0.022   0.552   0.426
E Y  4KROA                586    0.412  88.023  -0.952   0.021   0.451   0.528
E A  4KROA                587    0.223  24.531  -1.277   0.020   0.205   0.775
E D  4KROA                588    0.472  67.943  -1.379   0.018   0.088   0.893
E A  4KROA                589    0.647  71.354  -0.251   0.018   0.047   0.935
E G  4KROA                590    0.666  52.383  -0.245   0.005   0.015   0.979
E H  4KROA                591    0.464  84.474   0.704   0.005   0.015   0.979
E V  4KROA                592    0.425  65.292   1.805   0.022   0.359   0.619
B C  4KROA                593    0.036   5.026  -2.014   0.004   0.616   0.381
E H  4KROA                594    0.429  77.962   1.204   0.003   0.718   0.279
E L  4KROA                595    0.566 103.635   1.482   0.022   0.552   0.426
B C  4KROA                596    0.109  15.247  -0.056   0.019   0.141   0.840
E H  4KROA                597    0.520  94.661   0.538   0.018   0.019   0.964
E P  4KROA                598    0.660  93.668  -0.125   0.016   0.005   0.979
E N  4KROA                599    0.538  78.778   0.663   0.018   0.019   0.964
B C  4KROA                600    0.079  11.092  -1.169   0.052   0.084   0.864
E T  4KROA                601    0.475  65.910   0.435   0.056   0.142   0.802
E Y  4KROA                602    0.513 109.714   0.419   0.064   0.216   0.721
E G  4KROA                603    0.382  30.040  -0.187   0.064   0.216   0.721
B C  4KROA                604    0.064   9.014  -1.512   0.021   0.279   0.699
E T  4KROA                605    0.617  85.564  -0.542   0.004   0.197   0.799
E G  4KROA                606    0.435  34.203  -1.048   0.005   0.045   0.951
E P  4KROA                607    0.545  77.279  -1.401   0.005   0.015   0.979
E G  4KROA                608    0.566  44.568  -0.339   0.003   0.003   0.994
E L  4KROA                609    0.554 101.419  -0.218   0.003   0.003   0.994
E E  4KROA                610    0.690 120.543  -0.018   0.003   0.003   0.994
E G  4KROA                611    0.504  39.696   1.451   0.018   0.019   0.964
B C  4KROA                612    0.058   8.129  -1.812   0.018   0.088   0.893
E P  4KROA                613    0.411  58.335   0.566   0.020   0.205   0.775
E T  4KROA                614    0.549  76.132   1.130   0.020   0.205   0.775
B N  4KROA                615    0.082  11.961  -0.287   0.019   0.141   0.840
E G  4KROA                616    0.542  42.624   0.636   0.018   0.047   0.935
E P  4KROA                617    0.654  92.788   0.014   0.018   0.019   0.964
E K  4KROA                618    0.564 115.933  -1.364   0.058   0.017   0.925
B H  4KROA                619    0.296  53.842   0.401   0.052   0.084   0.864
B H  4KROA                620    0.299  54.297  -0.001   0.069   0.386   0.545
E H  4KROA                621    0.354  64.356   0.661   0.074   0.484   0.442
E H  4KROA                622    0.400  72.815  -0.963   0.069   0.386   0.545
E H  4KROA                623    0.412  74.961  -0.595   0.064   0.216   0.721
E H  4KROA                624    0.661 120.309  -1.982   0.003   0.003   0.994

Explain the output.  Go back.
```

---
